# AcnSP – A Novel Small Protein Regulator of Aconitase Activity in the Cyanobacterium *Synechocystis* sp. PCC 6803

**DOI:** 10.3389/fmicb.2020.01445

**Published:** 2020-06-26

**Authors:** Luna V. de Alvarenga, Wolfgang R. Hess, Martin Hagemann

**Affiliations:** ^1^Department of Plant Physiology, Institute of Biosciences, University of Rostock, Rostock, Germany; ^2^Genetics & Experimental Bioinformatics, Faculty of Biology, University of Freiburg, Freiburg im Breisgau, Germany; ^3^Department Life, Light and Matter, University of Rostock, Rostock, Germany

**Keywords:** cyanobacteria, central metabolism, metabolomics, mutant, small proteins, tricarboxylic acid cycle

## Abstract

*Synechocystis* sp. PCC 6803 is a widely used model cyanobacterium whose genome has been well annotated. However, several additional small protein coding sequences (sORFs) have been recently identified, which might play important roles, for example in the regulation of cellular metabolism. Here, we analyzed the function of a sORF encoding a 44 amino acid peptide showing high similarity to the N-terminal part of aconitase (AcnB). The expression of the gene, which probably originated from a partial gene duplication of chromosomal *acnB* into the plasmid pSYSA, was verified and it was designated as *acnSP*. The protein-coding part of *acnSP* was inactivated by interposon mutagenesis. The obtained mutant displayed slower growth under photoautotrophic conditions with light exceeding 100 μmol photons m^–2^ s^–1^ and showed significant changes in the metabolome compared to wild type, including alterations in many metabolites associated to the tricarboxylic acid (TCA) cycle. To analyze a possible direct impact of AcnSP on aconitase, the recombinant *Synechocystis* enzyme was generated and biochemically characterized. Biochemical analysis revealed that addition of equimolar amounts of AcnSP resulted in an improved substrate affinity (lower *K*_m_) and lowered *V*_max_ of aconitase. These results imply that AcnSP can regulate aconitase activity, thereby impacting the carbon flow into the oxidative branch of the cyanobacterial TCA cycle, which is mainly responsible for the synthesis of carbon skeletons needed for ammonia assimilation.

## Introduction

Cyanobacteria represent the only prokaryotes performing oxygenic photosynthesis. It is believed that this process was transferred into eukaryotic phototrophs such as algae and plants via an endosymbiotic uptake of a unicellular cyanobacterium as ancestor of plastids (Hohmann-Marriott and Blankenship, 2011; Ponce-Toledo et al., 2017). Due to their metabolic proximity to plants and easy genetic manipulation, cyanobacteria are popular models to study the molecular features of photosynthetic processes. More recently, cyanobacteria have come into focus as green cell factories that can be used as platforms for the CO_2_-neutral production of diverse valuable products (e.g., Hagemann and Hess, 2018).

One of the most frequently used cyanobacterial models is the strain *Synechocystis* sp. PCC 6803 (hereafter *Synechocystis* 6803). This strain is able to grow photoautotrophic, mixotrophic or heterotrophic due to its capability to use external glucose in addition to CO_2_ as carbon source (Rippka et al., 1979). Its wide-spread application was further supported by the discovery of natural competence for DNA uptake and incorporation (Grigorieva and Shestakov, 1982), which facilitated the development of highly efficient genetic systems. Finally, the genome of *Synechocystis* 6803 was completely sequenced already 25 years ago (Kaneko et al., 1996), which tremendously accelerated the functional analysis of diverse genes and proteins in this model strain. Meanwhile, multiple independent genome sequences of *Synechocystis* 6803 substrains became available (e.g., Trautmann et al., 2012). The genome of *Synechocystis* 6803 comprises one large chromosome and up to 7 plasmids of different sizes (Kaneko et al., 2003), which harbor annotated open reading frames (ORFs) encoding for approximately 3700 different proteins. However, recent transcriptome analyses applying different types of RNAseq that allowed precise mapping of transcriptional start sites revealed a much higher coding capacity, since many transcripts from alternative start sites as well as small non-protein-coding RNAs and antisense RNAs were detected (Mitschke et al., 2011; Kopf et al., 2014). These results indicated that even the well annotated and characterized genome of *Synechocystis* 6803 harbors more genes than initially assumed.

Recently, it has been discussed that many genome annotations are incomplete, since the traditionally applied thresholds for ORF predictions regularly missed the potential for encoding small proteins, even among bacteria. This problem became also obvious, when researchers discovered small ORFs (sORFs) on many sRNAs, which were initially believed to be non-protein-coding, regulatory RNAs (reviewed in Storz et al., 2014). The ongoing identification and characterization of such small proteins showed that they do not only exist but fulfill important regulatory functions for cell differentiation or division, transport activities, and protein kinases, respectively. Other small proteins from bacteria act as chaperones, signal molecules in interspecies communications, toxins or stabilizers of larger protein complexes (Hobbs et al., 2011; Storz et al., 2014). In *Synechocystis* 6803, many sORFs were already annotated, since they encode important and well-conserved subunits of protein complexes involved in photosynthesis and respiration (summarized in Baumgartner et al., 2016). During the last years, several additional small proteins have been functionally characterized, for example as cyanobacteria-specific subunits of the photosynthetic complex I (also known as NDHI complex), which is involved in photoheterotrophic growth, cyclic electron flow, and CO_2_ uptake (e.g., Zhang et al., 2004; Schwarz et al., 2013b; Wulfhorst et al., 2014; Schuller et al., 2019).

Baumgartner et al. (2016) used comparative genomics and transcriptomics to search for additional transcribed sORFs in *Synechocystis* 6803. They predicted 293 sORFs for proteins ≤80 amino acids, among them many newly annotated ones. Using a C-terminal tagging strategy, translation was demonstrated for 5 sORFs on the main chromosome implying that these small proteins not only exist but may be functionally relevant for *Synechocystis* 6803 (Baumgartner et al., 2016). One of the predicted but not previously validated sORFs is located on the plasmid pSYSA and potentially encodes a 44 amino acid small protein showing high similarities with the N-terminus of cis-aconitate hydratase (aconitase B, gene *acnB*, locus *slr0665* encoding the 868 amino acids long enzyme). We wondered if this sORF would merely be a pseudogenized gene fragment of the chromosomal *acnB* gene or could it be of some functional importance? The enzyme aconitase participates in the tricarboxylic acid (TCA) cycle that plays an important role for primary metabolism in organisms from all branches in the tree of life. Among cyanobacteria, the TCA cycle is supposed to be largely open, because the 2-oxoglutarate (2-OG) dehydrogenase complex is missing. Only recently, shunts potentially closing the cyanobacterial TCA cycle have been identified; however, the flux through these shunts appears to be rather low under most conditions (Zhang and Bryant, 2011; Xiong et al., 2014). Hence, the oxidative TCA branch initiated by aconitase is mainly responsible for the production of 2-OG, the precursor of ammonia assimilation among cyanobacteria. It has also been shown that the relative flux into the TCA cycle of cyanobacteria changes in dependence from growth conditions, for example the availability of inorganic carbon or nitrogen (Schwarz et al., 2013a). Recently, it has been reported that citrate synthase of the *Synechocystis* 6803 TCA cycle is highly regulated and that the TCA cycle intermediate citrate regulates the activity of the oxidative pentose-phosphate (OPP) pathway (Ito et al., 2019; Ito and Osanai, 2020).

Here, we analyzed if the predicted 44 amino acids short protein originating from plasmid pSYSA would play a role, especially in TCA cycle regulation. Indeed, we found that this small protein can improve the substrate affinity of aconitase. Accordingly, this gene was renamed as *acnSP* for aconitase small protein. Furthermore, these results demonstrate that sORFs can originate even from seemingly irregular gene fragment duplications and add another element to the complex regulatory system around AcnB.

## Materials and Methods

### Culture Conditions

The *Synechocystis* 6803 substrain M (Trautmann et al., 2012) was used in the study. The wild type (WT) and mutant strains were cultivated in glass Erlenmeyer flasks (125 ml) filled with 40 ml of buffered BG-11 medium (pH 8.0) without sodium carbonate (Rippka et al., 1979) under the following conditions: 28 ± 2°C and continuous light of 50 μmol photons m^–2^ s^–1^. The growth experiments were conducted at 30°C in a Multi-Cultivator MC1000-OD (Photon Systems Instruments, Czech Republic) with continuous illumination of 50 or 100 μmol photons m^–2^ s^–1^ and bubbling with ambient air. The MC1000-OD system performs a continuous recording of growth and pigmentation by measuring the optical density at 720 nm and 680 nm, respectively (here every 10 min data points). During the preculture, cells were grown in 50 ml BG11 medium aerated with 5 % (v/v) CO_2_-supplemented air in 350 ml column photobioreactors (580 × 30 mm) at 30°C under constant warm-white-light illumination of 100 μmol photons m^–2^ s^–1^. Mutant strains were grown in the presence of the respective antibiotics (50 μg ml^–1^ kanamycin or 10 μg ml^–1^ gentamycin) during the precultivation and without antibiotics during experiments for an accurate comparison with the WT. Pigmentation of strains was characterized by *in vivo* absorption measurements and calculated using the correction factors given by Sigalat and de Kouchkovsky (1975). The sampling for pigmentation and metabolome analysis was done from growing cultures 48 h after inoculation. Photographs from representative cultures were taken at the same time point.

### Construction of the Mutant Δ*acnSP* and Expression Strain pVZ322_AcnSP

The kanamycin resistance gene *aphII* was excised from pUC4K using *Bam*HI. The flanking sequences (500 bp in each direction) of the *acnSP* gene (nt positions 30142-30011 on plasmid pSYSA, accession number AP004311) were obtained from total DNA of the *Synechocystis* 6803 WT via PCR using the primers 1853p/1856p (Supplementary Table S1). Overhanging *Bam*HI or *Bam*HI/*Nde*I sites were created in the respective 5′ and 3′ flanking regions. The resulting sticky ends were ligated with the isolated *aphII* gene in the backbone of plasmid pGEM-T (Promega). The plasmid pG_acnSP_5F/3F:Km was transformed into WT cells of *Synechocystis* 6803, and clones were selected by supplementation of the medium with kanamycin. Subsequent cultivation at 50 μg ml^–1^ kanamycin resulted in complete segregation of the Δ*acnSP* mutant.

To generate the expression strain, the WT *acnSP* gene including 500 bp of the upstream native promoter sequence was obtained via PCR with the primers 2109p and 2103p that also permitted to add the FLAG-tag onto the 3′ end of the sORF (Supplementary Table S1). The PCR fragment was first inserted into pGEM-T (Promega). Inserts showing correct sequences were cleaved off with *Xho*I/*Pst*I and cloned into the plasmid pVZ322 (Zinchenko et al., 1999) treated with the same enzymes. *Escherichia coli* strains DH5α and RP4 were used for conjugation of the final vector into the WT, leading to strain pVZ322_AcnSP. Selection of clones was done with 10 μg ml^–1^ gentamycin.

### Metabolome Analysis

Samples of 5 ml each were taken from the cultures 48 h after inoculation. At this time point, the cell suspension had an optical density at 720 nm (OD_720_) of about 1, which according to our calibration curves is equal to approximately 5 × 10^7^ cells or 1.8 mg cell dry matter per sample. Cells were harvested by quick filtration (30 sec) on nitrocellulose filters (0.45 μm pore size, Sartorius, Germany). The filters were put in 2 ml Eppendorf tubes and immediately frozen in liquid nitrogen. Low molecular mass compounds were extracted from the cells with 2 ml of ethanol (80%, HPLC grade, Roth, Germany) at 65 °C for 2 h. One microgram of carnitine was added per sample as an internal standard. After centrifugation, the supernatants were collected and freeze-dried. The dry extracts were dissolved in 200 μl MS-grade water and filtered through 0.2 μm filters (Omnifix^®^-F, Braun, Germany). The cleared supernatants were analyzed using the high-performance liquid chromatograph mass spectrometer system (LCMS-8050, Shimadzu, Japan). In brief, 1 μl of each extract was separated on a pentafluorophenylpropyl (PFPP) column (Supelco Discovery HS FS, 3 μm, 150 × 2.1 mm) with a mobile phase containing 0.1% formic acid. The compounds were eluted at a rate of 0.25 ml min^–1^ using the following gradient: 1 min 0.1% formic acid, 95% distilled water, 5% acetonitrile, within 15 min linear gradient to 0.1% formic acid, 5% distilled water, 95% acetonitrile, 10 min 0.1% formic acid, 5% distilled water, 95% acetonitrile. Aliquots were continuously injected in the MS/MS part and ionized via electrospray ionization (ESI). The compounds were identified and quantified using the multiple reaction monitoring (MRM) values given in the LC-MS/MS method package and the LabSolutions software package (Shimadzu, Japan). The metabolites were determined as relative metabolite abundances, which were calculated by normalization of signal intensity to that of the internal standard carnitine and OD_720_.

Glycogen was quantified as described by Kirsch et al. (2017). Briefly, low molecular mass compounds were removed by ethanolic extraction. Glycogen in the cell pellet was than hydrolyzed by α-amylase to glucose, which was enzymatically quantified.

### Isocitrate Quantification via an Enzyme Assay

The isocitrate quantification was performed enzymatically, since the LC-MS/MS method is not distinguishing citrate and isocitrate. The assay is based in the conversion of isocitrate to 2-OG by isocitrate dehydrogenase (IDH) measuring the amount of produced NADPH (Siebert, 1963).

Isocitrate was quantified in the same extracts as used before for LC-MS/MS analyses. The reaction mixture contained 50 mM Tris-malate buffer (pH 7.5), 0.35 mM NADP^+^, 1 unit (12.9 μg/μL) IDH (Sigma-Aldrich, United States), 2 mM Na_2_EDTA, 10 mM MnCl_2_. Following the addition of 2 μl cell extract to the reaction mixture, the total increase in absorbance at 340 nm was monitored by a microplate reader (Synergy HTX, BioTek) at 30°C. The total volume of the reaction mixture was 200 μL. The calibration curve revealed a linear relation between isocitrate (Serva – Heidelberg, Germany) concentrations of 0.40–480 nmol with final NADPH absorption at 340 nm (*y* = 0.764*x* + 0.0004, *R*^2^ = 0.9989, *y* isocitrate concentration, ×ΔOD340 nm).

### Generation of Recombinant Aconitase in *E. coli*

Recombinant AcnB protein was generated for biochemical analysis. The WT *acnB* gene was obtained from *Synechocystis* 6803 DNA via PCR with the primer pair 2075p/2095p (Supplementary Table S1). The fragment was cut with *Nde*I/*Sal*I and ligated into plasmid pET28a(+) (Novagen) cleaved with the same enzymes yielding plasmid pET28_*acnB*. After transformation into *E. coli*, clones carrying the recombinant plasmid were selected at 50 μg ml^–1^ kanamycin. The integrity of the vector was verified by plasmid DNA preparation, restriction and sequence analyses. AcnB protein was obtained in soluble form after expression in *E. coli* strain BL21 (DE3) harboring the vector pET28_*acnB*. To ensure incorporation of iron-sulfur centers, the LB medium was supplemented with an additional amount of Fe-citrate (final concentration of 0.03 mM). The His-tagged recombinant AcnB protein was purified from lysates using affinity chromatography on Ni-NTA columns (ProBond^TM^ resin, Life Technologies). To this end, frozen *E. coli* cells were suspended in 5 ml of 20 mM Tris buffer (pH 8) containing 300 mM NaCl and proteins were extracted by sonication (2-times 60 s on ice). Cell debris was separated from the lysate by centrifugation (20,000 × *g*, 4°C). The supernatant with soluble proteins was loaded on equilibrated Ni-NTA columns containing about 3 ml of the affinity matrix. The 20 mM Tris buffer (pH 8) containing 300 mM NaCl buffer was used for equilibration of affinity columns and the first two washing steps (each done with 10 ml of buffer). The third washing buffer contained 80 mM imidazole to remove non-specifically bound proteins. Bound protein was then eluted 3-times with each 1 ml of 20 mM Tris buffer (pH 8) containing 300 mM imidazole (elution fractions 1–3). Protein profiles from a typical purification procedure are shown in the Supplementary Figure S1. The elution fraction one was used for activity measurements.

### Protein Analysis by Polyacrylamide Gel Electrophoresis and Western-Blotting

Proteins were usually separated by standard SDS-PAGE (12% acrylamide) using the minigel system (Bio-Rad). Proteins were denatured by 5 min boiling in Laemmli-buffer containing 70 mM SDS and 2.4 mM 2-mercaptoethanol. The separated proteins were either stained with Coomassie-brilliant blue or transferred onto PVDF membranes (Hybond, Amersham) using electro-blotting for subsequent protein detection with specific antibodies.

Soluble protein extracts (10 μg) from cells of WT, Δ*acnSP*, or pVZ322_AcnSP, which were cultivated at 100 μmol photons m^–2^ s^–1^ of continuous light, were used for Western-blotting. For the detection of aconitase a peptide antibody was generated. The synthetic peptide (Ac)-CELLKNPPEAKEEL-amidated specific for AcnB of *Synechocystis* 6803 was used to produce antiserum in rabbits (Agrisera AB, Sweden). The FLAG-tagged AcnSP protein version in strain pVZ322_AcnSP was detected with a commercial anti FLAG-tag antibody [Monoclonal ANTI-FLAG M2-Peroxidase (HRP), Sigma-Aldrich, United States].

### Characterization of Aconitase Activity

Aconitase activity was measured using a coupled enzyme assay. The assay involves the conversion of cis-aconitate by aconitase into isocitrate followed by its oxidation to 2-OG via IDH with concomitant recording of the reduction of NADP^+^ to NADPH at 340 nm.

The reaction mixture contained 50 mM Tris-malate buffer (pH 7.5), 0.3 mM NADP^+^, 1 unit IDH (Sigma-Aldrich, United States), 2 mM Na_2_EDTA, 10 mM MnCl_2_ and purified AcnB protein (2 nmol). The reaction was started by the addition of different amounts of cis-aconitate (0.02–5.7 mM) to the reaction mixture. The enzyme activity assay comprised a final volume of 200 μl in wells of a UV-transparent microtiter plate. The increase in absorbance at 340 nm was monitored by a microplate reader (Synergy HTX, BioTek) for 30 min at 30°C. To investigate the effect of the peptide AcnSP on the AcnB activity, 2 nmol of the peptide [dissolved in water; synthesized by JPT (Germany)] was added to the reaction mix. A similar amount of the synthetic peptide Norf1 (Baumgartner et al., 2016), synthesized by the same manufacturer, was used in assays as negative control. The activity was measured under reducing conditions (with 10 mM DTT) or oxidizing condition (without added DTT). The substrate-dependent aconitase activity followed Michaelis-Menten kinetics. The kinetic parameters of aconitase were calculated using a Lineweaver-Burk plot. The equation slope is the *K*_m_/*V*_max_. The intercept on the vertical axis is 1/*V*_max_, while the intercept on the horizontal axis represents −1/K_m_ (Berg et al., 2002).

### Statistical Analysis

All experiments were performed with three biological replicates (three independent culivations or three independent protein expressions) and each two technical replicates. The presented data are means and standard deviations (*n* = 6). Statistical analysis was done using Software R studio.

## Results and Discussion

### *In silico* Analysis and Expression Verification of AcnSP

The sORF encoding AcnSP was initially predicted as ORF3 on the *Synechocystis* 6803 plasmid pSYSA (therefore originally assigned as pSYSA_ORF3, position 30008 to 30142; Baumgartner et al., 2016). This sORF was annotated using the program RNAcode (Washietl et al., 2011). RNAcode predicts local regions of high coding potential on the basis of multiple sequence alignments and the evolutionary signatures that are associated with a coding sequence together with an estimate of statistical significance in the form of a *P*-value. The cut-off for a given sequence to be considered as protein-coding is a *P*-value ≤ 0.001 in *Drosophila*, ≤0.05 in bacteria (Washietl et al., 2011). The *P*-value for the prediction of *acnSP* as protein-coding was 4 × 10^–7^ (Baumgartner et al., 2016). Hence, it was considered as a high-ranking candidate for further analysis.

**FIGURE 1:**
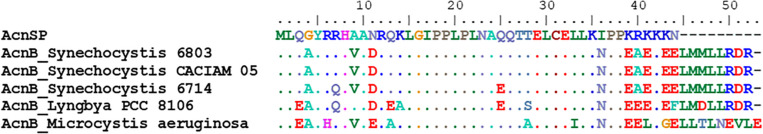
Protein alignment of AcnSP with the N-terminal fragment of AcnB. The 50 amino acids of the N-terminus of the most similar AcnB proteins from cyanobacterial strains were compared with the sequence of AcnSP. Dots indicate identical amino acids in all six sequences at the respective position.

**FIGURE 2:**
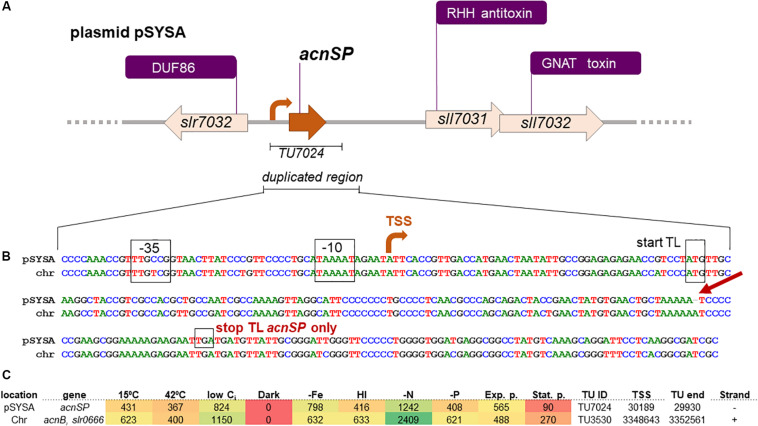
Details on *acnSP* sequence and regulatory elements in *Synechocystis* 6803. **(A)** The gene *acnSP* is located in an intergenic region of plasmid pSYSA, upstream of a toxin-antitoxin system that consists of a ribbon-helix-helix (RHH) domain-containing antitoxin and a GCN5-related N-acetyltransferase (GNAT) domain-containing toxin (Kopfmann and Hess, 2013). According to the previous genome-wide mapping of transcriptional start sites (TSS), it is transcribed as part of a monocistronic transcriptional unit (TU7024) with a TSS at position 30189 on the reverse strand (here the orientation has been reversed for simplification). **(B)** DNA sequence alignment of the duplicated region in pSYSA encompassing *acnSP* with the corresponding chromosomal region (chr) containing the promoter and begin of *acnB*. The conserved promoter elements, TSS, and the start of translation are indicated. The single nucleotide deletion leading to the truncation of the *acnSP* reading frame after the 36th codon is indicated by the red arrow. **(C)** Heatmap indicating the expression of *acnSP* (top) and of the *acnB-slr0666* dicistron (lower line) under 10 different growth conditions: exponential (Exp.) and stationary growth phase (Stat.); cold (15°C) and heat (42°C) stress; depletion of inorganic carbon (low C_i_); dark; iron depletion (–Fe); high light (HL); nitrogen depletion (–N); phosphate depletion (–P). The data were derived from previous genome-wide expression analysis by differential RNA-Seq performed by Kopf et al. (2014), which also contains further experimental details. Values indicate sequencing read counts for the primary 5′ end (=TSS) of the corresponding transcriptional unit (TU) after scaling to library sizes of 100 million reads, followed by normalization with a library-specific correction factor for the fraction of TSS-associated read counts (Kopf et al., 2014). The TSS positions are given for the *Synechocystis* 6803 chromosome and plasmid pSYSA available under accession numbers BA000022 and AP004311. The color varies from red (no expression) to yellow (intermediate expression) to green (high expression).

The gene *acnSP* analyzed in this work encodes a 44 amino acid peptide that is 81% identical to the N-terminal part of AcnB from *Synechocystis* 6803. The sequences deviate from each other at only four positions within the first 38 amino acids, while five of the six last residues are different due to a single nucleotide deletion in the 36th *acnSP* codon compared to *acnB* (Figures 1, 2).

Previous analysis of the *E. coli* AcnB structure revealed that the approximately 160 amino acids long N-terminus forms the domain 5, which is absent from mitochondrial aconitases comprising only four domains (Williams et al., 2002). This part folds as HEAT-like domain, which consist of four antiparallel α-helix pairs in the AcnB protein of *E. coli*. Hence, the only 44 amino acid long AcnSP peptide might be able to form one pair of antiparallel α-helices as predicted by an *in silico* protein folding program (Supplementary Figure S2). The presence of the HEAT-like domain 5 in AcnB-type aconitases differentiate these from bacterial AcnA and mitochondrial aconitases (Tang et al., 2005). The initial structural analysis implied a monomeric AcnB (Williams et al., 2002); however, subsequent analysis showed that bacterial AcnB usually forms a homodimer that shows higher enzymatic activity than the monomeric AcnB (Tang et al., 2005). HEAT-like protein domains are known to be involved in protein/protein interactions. Deletions of the N-terminal part of bacterial AcnB verified that the HEAT-like domain is relevant for homodimer formation (Tang et al., 2005) or might facilitate interaction of the monomeric AcnB protein with other enzymes of the TCA cycle to allow substrate channeling (Williams et al., 2002). Hence, we reasoned if AcnSP could interfere with AcnB dimer formation or the interaction of aconitase with other proteins.

A closer inspection of the DNA sequence revealed that a fragment of the *acnB* gene was transferred from the chromosome into pSYSA, since in addition to the well conserved sORF highly similar DNA sequences were also observed upstream, comprising the putative promoter region, as well as downstream of *acnSP* (Figure 2). This gene duplication then allowed the accumulation of point mutations within the plasmid gene copy, which affected the protein-coding sequence leading to the appearance of the observed amino acids exchanges and a stop codon after the 44th codon. Hence, *acnSP* on plasmid pSYSA encodes a strongly truncated version of the chromosomally encoded aconitase AcnB (Slr0665). This plasmid has a size of 100,749 bp in the substrain PCC-M of *Synechocystis* 6803 (Trautmann et al., 2012). Previous work characterized plasmid pSYSA for its defense-related functions as it harbors three different, complete CRISPR-Cas systems (Scholz et al., 2013) and at least seven different toxin-antitoxin systems (Kopfmann and Hess, 2013). Our work suggests that with *acnSP* additional functions are linked to pSYSA.

Expression of the *acnSP* gene on pSYSA was detected in multiple microarray data sets (e.g., Klähn et al., 2015), but due to the high similarity on DNA level these signals could also have originated from the *acnB* expression instead of *acnSP*. However, mapping the reads from differential RNA-Seq analysis (Kopf et al., 2014) with stringent parameters unequivocally demonstrated that *acnSP* is transcribed in the form of a 260 nt long transcriptional unit (TU7024) with a TSS at position 30189 on the reverse strand of pSYSA. The upstream promoter elements of *acnSP* and *acnB* are highly conserved (Figure 2B), which is consistent with their similar regulation, i.e., maximum expression under low nitrogen conditions and low to no detectable expression in stationary phase cultures or during darkness (Figure 2C).

**FIGURE 3:**
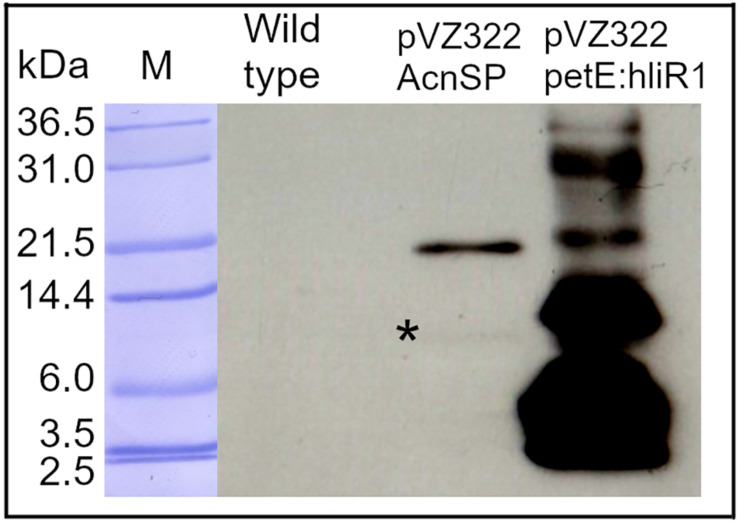
Verification of *acnSP* expression. A FLAG-tagged version of *acnSP* was expressed in strain pVZ322_AcnSP from its native promoter. 10 μg of protein were applied onto the SDS gel and FLAG-tagged proteins were detected with a commercially available antiserum. A specific signal was observed in the extracts of strain pVZ322_AcnSP but not in WT. Protein extracts of strain pVZ322-petE:hliR1 accumulating a FLAG-tagged version of the small protein HliR1 (Baumgartner et al., 2016) were included as positive control (M-protein size marker Mark12^TM^ Unstained Standard Ladder, Thermo Fisher Scientific, *marks the weak signal around 10 kDa recognized by the FLAG-tag antibody).

Next, we aimed to verify the expression of the *acnSP* gene on protein level. To this end, we expressed a FLAG-tagged version of *acnSP* from the autonomously replicating plasmid pVZ322, on which it is was cloned under the control of its native promoter. The FLAG-tag antibody detected a specific signal in protein extracts of the strain pVZ322_AcnSP, whereas no signal was obtained with total protein extracts from the WT (Figure 3). The main signal was detected at about 20 kDa and only a very faint signal is visible at the expected size of approximately 10 kDa (calculated molecular mass of AcnSP-FLAG is 8.08 kDa). The HliR1 protein used as positive control is a 37 amino acids small protein of unknown function. Together with the triple FLAG tag, HliR1 has a calculated molecular mass of 6.93 kDa but runs in SDS-PAGE, similar to many other small proteins, at a higher apparent molecular mass probably originating from multimers (Baumgartner et al., 2016). This behavior was reproduced here for HliR1 and noticed for FLAG-tagged AcnSP as well, which has been mainly detected at approximately 20 kDa even under denaturating conditions in the Western-blotting experiments (Figure 3) pointing at the formation of relatively stable AcnSP dimers. These experiments verified that the *acnSP* gene was expressed in *Synechocystis* 6803 on protein level.

### An *acnSP* Mutant Reveals a High Light-Sensitive Phenotype

To obtain information about the function of the expressed AcnSP protein, we generated the Δ*acnSP* mutant by interposon mutagenesis. A kanamycin-resistance cartridge was inserted into the *acnSP*-coding sequence. The WT-gene copy on the plasmid pSYSA was completely replaced, since only fragments specific for the mutated gene copy were detected during genotyping via PCR (Supplementary Figure S3).

**FIGURE 4:**
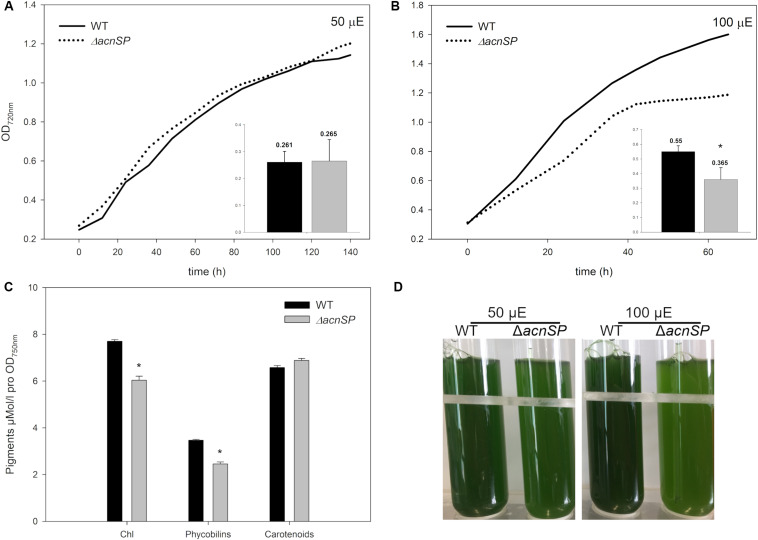
Characterization of mutant Δ*acnSP*. **(A)** Growth experiment with the WT and the mutant grown under continuous light of 50 μE or **(B)** 100 μE. The growth as increase in OD_720_ is shown from one representative experiment. The inserts show calculated growth rates per day of the WT (black columns) and the mutant (gray columns) (mean and standard deviation from three growth experiments). **(C)** Estimation of pigment contents in cells of the WT and mutant Δ*acnSP* grown at 100 μE continuous light. **(D)** Appearance of the WT and mutant Δ*acnSP* grown at 50 or 100 μE continuous light for 48 h. The asterisks mark statistically significant differences between the WT and mutant (Student’s *t*-test, *P*-value < 0.01).

Then, cells of the mutant Δ*acnSP* were grown under different culture conditions. The strain showed growth like WT under low continuous light of 50 μmol photons m^–2^ s^–1^ (50 μE) (Figure 4A), but also under other tested growth conditions such as light/dark changes, presence of high CO_2_ (5%) or ambient CO_2_ conditions (0.04%), as well as growth in the presence of glucose under light or dark conditions (data not shown, always at 50 μE). However, it showed slower growth under higher light intensities such as 100 μmol photons m^–2^ s^–1^ (100 μE) (Figure 4B). Interestingly, the Δ*acnSP* mutant also displayed a slight change in the pigmentation pattern when grown at continuous light of 100 μE. Mutant cells appeared more yellow-greenish due to the reduced amount of phycocyanin and chlorophyll (Figures 4C,D), which may indicate changes in the N-assimilation as particularly phycocyanin also serves as N-storage protein (e.g., Klotz et al., 2016). The pigmentation phenotype was absent under low light conditions.

### Metabolic Analysis of the Δ*acnSP* Mutant

The observed high light-sensitive phenotype of the Δ*acnSP* mutant could be due to the involvement of AcnSP in regulating the metabolism of *Synechocystis* 6803, which is also expected due to its high similarity with AcnB. Hence, differences in carbon as well as nitrogen assimilation might exist due the potential impact of AcnSP on TCA cycle activity. We applied targeted metabolomics using LC-MS/MS to receive a snapshot of steady state values of 27 cellular metabolites (Supplementary Table S2). Many changes in the abundance of intermediates of the central carbon and nitrogen metabolism were detected, which indicate that AcnSP might be involved in metabolic regulation in *Synechocystis* 6803.

**FIGURE 5:**
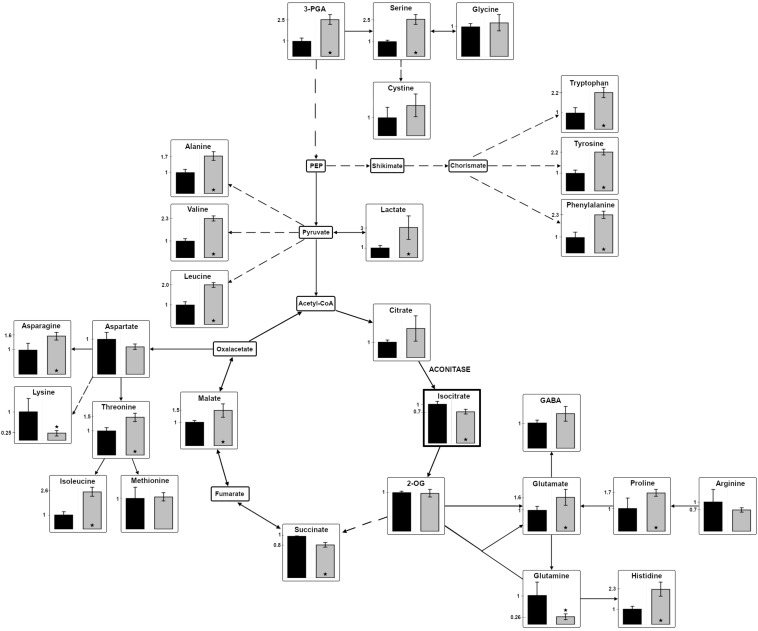
Changes in the relative abundances of metabolites in the mutant Δ*acnSP*. Polar low molecular mass metabolites were isolated from cells of the mutant Δ*acnSP* or the WT, which were cultivated at continuous light of 100 μE for 48 h. Metabolites of the central carbon and nitrogen metabolism were quantified by LC-MS/MS in extracts of the mutant Δ*acnSP* (gray columns) or the WT (black columns) of *Synechocystis* 6803. Only isocitrate was quantified by an enzymatic assay (indicated by the thicker frame). The relative amount of the metabolite in WT cells was set to 1 and fold changes of mutant metabolite levels are displayed. The relative metabolite abundance can be found in the Supplementary Table S2. Broken arrows indicate that more than one enzymatic step is necessary for the conversion of certain metabolites. Asterisks indicate statistically significant differences (Student’s *t*-test, *P*-value ≤ 0.05) (2-OG – 2-oxoglutarate; 3PGA – 3-phosphoglycerate; GABA – γ-aminobutyric acid; PEP – phosphoenolpyruvate).

Interestingly, citrate, the substrate of aconitase accumulated to 2-fold higher levels in cells of mutant Δ*acnSP* than in the WT. Assuming a direct effect of AcnSP on aconitase activity, this finding could indicate that the absence of AcnSP negatively impacts aconitase. It should be noted that the applied LC-MS/MS method does not permit to distinguish between citrate and isocitrate due to their similar masses and identical retention times. It has been reported that the citrate pool is 2- to 5-times higher in *Synechocystis* 6803 (Takahashi et al., 2008; Dempo et al., 2014) as well as in *Microcystis aeruginosa* (Meissner et al., 2015). Therefore, the LC-MS/MS value was used as citrate measure. To quantify the specific amount of isocitrate, we used an enzymatic detection method with IDH. This method showed that the isocitrate amount was significantly reduced in extracts of the mutant Δ*acnSP* compared to WT (Δ*acnSP* 0.061 ± 0.003 μmol isocitrate OD_720_^–1^ ml^–1^ versus WT 0.084 ± 0.0032 μmol isocitrate OD_720_^–1^ ml^–1^, *n* = 6). In addition, many other intermediates of the TCA cycle also showed deviations between WT and mutant extracts. The amount of glutamine, the primary product of ammonia assimilation via the GS-GOGAT cycle, was approximately 4-fold lower in the Δ*acnSP* mutant, while the steady state amount of 2-OG remained unchanged (Figure 5). The reduced glutamine amount is consistent with the reduced pigmentation of Δ*acnSP*. Most other detected amino acids showed elevated levels in mutant extracts, but some of them, like lysine were lower in the mutant than in WT.

In addition to citrate, the malate content was also increased in mutant extracts, which might indicate that the presumably lowered carbon flux into the oxidative branch of the TCA cycle led to an enhanced flux into its reductive branch in the absence of AcnSP. Furthermore, the steady-state amount of 3-phosphoglycerate (3PGA), the primary carboxylation product of ribulose 1,5-bisphosphate carboxylase/oxygenase, was increased in mutant cells compared to WT regardless if grown at ambient air (Figure 5) or high CO_2_ conditions. The 3PGA accumulation could indicate that mutant cells show slower flux through the Calvin cycle, which results in reduced CO_2_ fixation compared to WT consistent with the slower growth of this strain. In line with this assumption is also the observation that cells of the mutant Δ*acnSP* accumulated approximately 40% less glycogen than WT (Δ*acnSP* 0.037 ± 0.005 mmol glucose OD_720_^–1^ ml^–1^ versus WT 0.0638 ± 0.0026 mmol glucose OD_720_^–1^ ml^–1^, *n* = 6).

It is surprising to see that the absence of AcnSP has such a broad impact on metabolite levels when grown at 100 μE. Many of these differences can be rather indirect effects of the slower growth of the Δ*acnSP* mutant. However, we suggest that AcnSP impacts aconitase activity, therefore, the changes in citrate and isocitrate levels may be directly linked to the presence or absence of this small peptide. It has recently been shown that citrate acts as potent inhibitor of the two entrance enzymes in the OPP pathway in *Synechocystis* 6803 (Ito and Osanai, 2020). Hence, citrate-induced changes in OPP activity might also contribute to the here observed differences in metabolite levels in cells of the Δ*acnSP* mutant compared to the WT.

Collectively, these results indicate that AcnSP plays a role in the regulation of central carbon and nitrogen metabolism at least under elevated light intensities, presumably due to its direct influence on aconitase activity. To rule out that the aconitase expression changes in cells of mutant Δ*acnSP*, the amount of aconitase protein was quantified in Western-blotting experiments with a specific antibody. We did not observe significant differences in aconitase abundance in protein extracts of the WT and Δ*acnSP* (Supplementary Figure S4), which supports a likely direct effect of AcnSP on aconitase activity further.

### Aconitase Activity and Impact of AcnSP

To investigate a possible direct impact of AcnSP on aconitase, we expressed the *Synechocystis* 6803 *acnB* gene in *E. coli* and obtained pure recombinant protein for biochemical investigations (Supplementary Figure S1). The recombinant aconitase from *Synechocystis* 6803 was obtained as enzymatically active protein from *E. coli* supplemented with extra amounts of iron to ensure proper incorporation of the iron-sulfur centers into aconitase. To obtain kinetic parameters, the aconitase activity was measured over a broad substrate range and Michaelis-Menten kinetics was found (Supplementary Figure S5). Its activity was significantly higher when tested under reducing than under oxidizing conditions as was previously reported for aconitase activity in plant extracts (Carrari et al., 2003). The increased aconitase activity in the presence of DTT could be also due to a stabilizing effect of reducing conditions on the iron-sulfur centers in AcnB as has been shown for aconitases from *E. coli* (Jordan et al., 1999). In the presence of very high amounts of cis-aconitate, the activity of aconitase started to decrease. Therefore, we evaluated only enzyme activities between 0.02 and 2 mM cis-aconitate in Lineweaver-Burk plots (Supplementary Figure S6). An about 4-fold higher *V*_max_ was observed under reducing conditions (presence of 10 mM DTT) than under oxidizing conditions, whereas the affinity toward cis-aconitate was higher under oxidizing conditions (lower *K*_m_, Table 1). To prove the impact of AcnSP on the kinetic parameters of aconitase, the enzyme assays were then supplemented with equimolar amounts of a synthetic AcnSP peptide. The addition of AcnSP to enzyme assays under oxidizing conditions as well as reducing conditions increased the affinity of aconitase for cis-aconitate, since in both cases the *K*_m_ values decreased (Table 1). We measured a K_m_ of 0.371 mM for aconitase in the presence of DTT, which decreased to 0.268 mM with AcnSP supplementation. According to the enzyme data base BRENDA, these *K*_m_ values are in the upper range of *K*_m_ values reported before for bacterial aconitase B proteins (https://www.brenda-enzymes.org/enzyme.php?ecno=4.2.1.3#KM%20VALUE%20[mM]) as well as the biochemical features of aconitases from *E. coli* (Jordan et al., 1999). In addition to the significantly decreased *K*_m_ values, we also measured an AcnSP-induced decline in *V*_max_ of aconitase (Table 1). To rule out non-specific effects of peptide addition, we performed aconitase activity tests in the presence of another synthetic small peptide Norf1 (Baumgartner et al., 2016). These tests were performed at substrate concentrations near the *K*_m_ values and showed no effect of the unrelated Norf1 peptide on aconitase activity (Supplementary Figure S7), supporting that the observed changes of aconitase substrate affinity were specific for AcnSP.

**TABLE 1:** Kinetic parameters of the *Synechocystis* 6803 aconitase.

	−DTT		+DTT	
	Control	AcnSP	Control	AcnSP
*V*_max_ (μmol mg^–1^ min^–1^)	2.10 ± 0.08	1.91 ± 0.25*	9.04 ± 0.13	8.34 ± 0.20*
*K*_m_ (mM)	0.051 ± 0.002	0.047 ± 0.005*	0.371 ± 0.020	0.268 ± 0.007*
*K*_cat_ (s^–1^)	3.46	3.15	14.92	13.76

*The aconitase activity was measured in the presence of different cis-aconitate concentrations. It showed Michaelis-Menten kinetics (see Supplementary Figure S5), which permitted to calculate the kinetic parameters *V*_max_, *K*_m_, and *K*_cat_ of aconitase under reducing (+10 mM DTT) or oxidizing (−DTT) conditions using a Lineweaver-Burk plot (see Supplementary Figure S6). Approximately 2 nmol recombinant *Synechocystis* 6803 aconitase was used per enzyme assay in the absence (control) or presence (AcnSP) of 2 nmol of the synthetic AcnSP peptide. The asterisks mark statistically significant differences between the WT and mutant (Student’s *t*-test, *P*-value < 0.05).*

The enzyme activity data showed that AcnSP has a significant impact on aconitase activity. It has the potential to positively influence the substrate affinity of aconitase. Hence, a lower affinity toward cis-aconitate in the absence of AcnSP is consistent with the accumulation of citrate in extracts of the mutant Δ*acnSP* (see Figure 5) pointing at a positive effect of AcnSP on citrate to isocitrate conversion especially under non-saturating substrate concentrations as can be expected *in vivo*.

This impact on the kinetic parameters is probably based on a direct interaction of the N-terminus of aconitase with AcnSP. It has been shown that the N-terminal part of bacterial aconitase proteins, which shows the similarities with AcnSP, folds into a HEAT-like domain, which is supposed to be crucially involved in protein/protein interactions. Such interactions may assist the formation of aconitase homodimers impacting its enzyme activity or heterodimers with other proteins of the TCA cycle, which has been proposed to achieve channeling of substrates through the enzymes of the TCA cycle (Williams et al., 2002; Tang et al., 2005).

## Conclusion

Collectively, our results suggest a role of the small protein AcnSP in the adjustment of *Synechocystis* 6803 central metabolism through aconitase activity regulation. This conclusion is based on the observed changes in the metabolome, in which the absence of AcnSP resulted in elevated citrate and reduced isocitrate levels but did not change the aconitase protein level. The interpretation of an AcnSP impact on aconitase activity was supported by the results of the *in vitro* enzyme assays, in which addition of AcnSP improved the substrate affinity of aconitase activity. Hence, the decreased substrate affinity is consistent with the higher citrate and lowered isocitrate levels in mutant cells. Furthermore, under oxidizing conditions, which are characteristic for cells under stress conditions such as high light, the aconitase activity is much smaller than under reducing conditions. The observed impact of AcnSP on aconitase probably originates from their direct interaction. It has been shown that the dimeric form of aconitase shows higher enzymatic activity in *E. coli* (Tang et al., 2005). It might be possible that AcnSP somehow interacts with the aconitase dimers and thereby influences the activity of the enzyme but not its amount. The rather broad changes of the metabolome in cells of the mutant Δ*acnSP* most probably result from indirect effects, such as the impact of changed citrate on OPP pathway activity (Ito and Osanai, 2020) or the general impact of slower growth on carbon and nitrogen fluxes (e.g., Jahn et al., 2018).

AcnB is the archetypical representative of a moonlighting protein with dual, enzymatic as well as regulatory functions in bacteria (Tang et al., 2005; Austin et al., 2015; Jeffery, 2014; Commichau and Stülke, 2015). AcnB-type aconitases of various organisms can switch between a metabolically active and a regulatory function by cycling between a dimeric and a monomeric state, respectively (Tang et al., 2005; Austin et al., 2015). Hence, aconitase not only plays an important role as enzyme in the TCA cycle, but its iron-free apo-form can also participate in the sensing of the iron status in bacterial and eukaryotic cells. Here, the N-terminal part of iron-free apo-aconitases is supposed to be involved in iron-mediated binding of RNAs. However, the HEAT-like domain of AcnB has almost no surface charge facilitating the interaction of this protein part with RNAs, which indicates that other AcnB domains are responsible for RNA binding (Williams et al., 2002). In this regard it is interesting to note that AcnSP includes 5 positively charged lysine and one arginine residue at its newly acquired C-terminal end, which could make this peptide particularly well suited for RNA binding. Furthermore, it has been shown that the iron stress response among cyanobacteria also affects iron-sulfur centers and induces the production of reactive oxygen species as it is also characteristic for high-light stress (Havaux et al., 2005; Georg et al., 2017). Hence, the high-light phenotype of mutant Δ*acnSP* could be taken as a first hint that AcnSP might also have additional functions in iron-sensing in addition to its verified role in the regulation of aconitase and thereby TCA cycle activity.

Given the facts that AcnB is the prototype of a moonlighting protein, an active enzyme in the TCA cycle and a regulator in the response to variations in iron concentrations, which itself is controlled by the post-transcriptional regulatory sRNA RyhB in *E. coli* (Massé et al., 2005) or IsaR1 in *Synechocystis* 6803 (Georg et al., 2017), we conclude that the here identified small protein AcnSP, which originates from incorporation of a fragment of the chromosomal *acnB* locus into the plasmid pSYSA, adds another element in the complex regulatory system around AcnB.

## Data Availability Statement

The raw data supporting the conclusions of this article will be made available by the authors, without undue reservation.

## Author Contributions

MH and LA designed the work. LA carried out most the experiments and evaluated the data together with MH. WH performed the bioinfomatic analyses and gave scientific input for improvement of the manuscript. MH edited and approved the final manuscript with input from all authors.

## Conflict of Interest

The authors declare that the research was conducted in the absence of any commercial or financial relationships that could be construed as a potential conflict of interest.
