# Supplementary material for: AcnSP – A Novel Small Protein Regulator of Aconitase Activity in the Cyanobacterium Synechocystis sp. PCC 6803
Source: Front Microbiol. 2020 Jun 26;11:1445. doi: 10.3389/fmicb.2020.01445 (PMC7336809; doi:10.3389/fmicb.2020.01445)
Supplement: Supplementary file 1 [file Data_Sheet_1.pdf]

SUPPLEMENTARY MATERIAL

Table S1: Primers used in this work

Code	Sequence	Region
**1853p**	TAAAAAATTTCTCCCCAAAGATTTAATTGTCT	5’ flanking region AcnSP_fw
**1856p**	CATATGACGACAAAATCAGTTAATGATCTGCC	3’ flanking region AcnSP_rv
**1759p**	GCTGGACGGTAACCGAGTTC	pVZ322::3xflag_rv
**2075p**	CATATGTTGCAAGCCTACCGTCG	aconitase_fw (*Nde*I)
**2095p**	GTCGACTTACTTAGCTCCCACTGGCA	aconitase_rv (*Sal*I)
**2103p**	CTGCAGCTATTTATCATCATCATCTTTATAATCAATATCAT GATCTTTATAATCGCCATCATGATCTTTATAATCATTCTT CTTTTTCCGCTT	AcnSP_rv_ FLAG-tag
**2109p**	CTC GAG TAA AAA ATT TCT CCC CAA AGA TTT	5’ flanking region AcnSP_fw (*Xho*I)

Supplementary Table S2: Relative amounts of metabolites of the central carbon and nitrogen metabolism, which were quantified by LC-MS/MS. The values represent compound-specific peak areas, which were normalized by the peak area of the internal standard carnitine and cell density measured as optical density at 720 nm and sample volume (relative area OD_{720}^{-1} mL^{-1}). The Table displays mean values and standard deviations (n=6). Metabolites were isolated from cells of the mutant ΔacnSP or the WT cultivated at continuous light of 100 µE for 48 h. Statistical significance was tested using Student's T-test ($p < 0.05$) and is indicated by asterisk.

Metabolites	**WT**	**ΔacnSP**	**Fold change**
**Aspartate**	819.2 ± 78.8	642.2 ± 31.4	0.78
**Serine**	139.5 ± 7.5	352.1 ± 22.9 *	2.52
**Alanine**	847.4 ± 60.8	1504.8 ± 87.9 *	1.78
**Glycine**	123.1 ± 6.0	139.5 ± 16.8	1.13
**Glutamine**	634.1 ± 147.2	165.9 ± 30.9 *	0.26
**Threonine**	216.6 ± 15.1	337.2 ± 17.8 *	1.56
**Glutamate**	10053.6 ± 946.3	16287.0 ± 1896.9 *	1.62
**Proline**	105.9 ± 24.1	177.6 ± 7.9 *	1.68
**Lysine**	689.3 ± 160.9	172.7 ± 33.7 *	0.25
**Arginine**	397.6 ± 83.7	288.2 ± 14.5	0.72
**Valine**	130.3 ± 9.5	306.3 ± 9.7 *	2.35
**Isoleucine**	114.4 ± 12.6	303.8 ± 18.8 *	2.66
**Leucine**	190.1 ± 17.2	390.3 ± 11.6 *	2.05
**Tyrosine**	65.2 ± 5.3	141.9 ± 5.0 *	2.18
**Methionine**	27.3 ± 5.1	28.9 ± 1.8	1.06
**Phenylalanine**	91.6 ± 15.0	223.8 ± 10.7 *	2.44
**Tryptophan**	22.1 ± 3.7	50.2 ± 3.4 *	2.27
**Citrate**	18.9 ± 1.2	35.1 ± 7.6	1.86
**Succinate**	8.5 ± 0.01	6.7 ± 0.2 *	0.79
**GABA**	0.03 ± 0.001	0.04 ± 0.004	1.38
**Cystine**	2.2 ± 0.6	3.7 ± 0.6	1.67
**Asparagine**	21.7 ± 3.0	34.2 ± 1.6 *	1.57
**Histidine**	7.6 ± 0.66	17.6 ± 1.7 *	2.30
**3-PGA**	2.8 ± 0.3	6.8 ± 0.4 *	2.45
**2-Oxoglutarate**	7.2 ± 0.1	7.1 ± 0.3	0.98
**Malate**	94.7 ± 3.5	141.4 ± 13.2 *	1.49
**Lactate**	16.4 ± 2.2	50.8 ± 9.8 *	3.10

Supplementary Figures

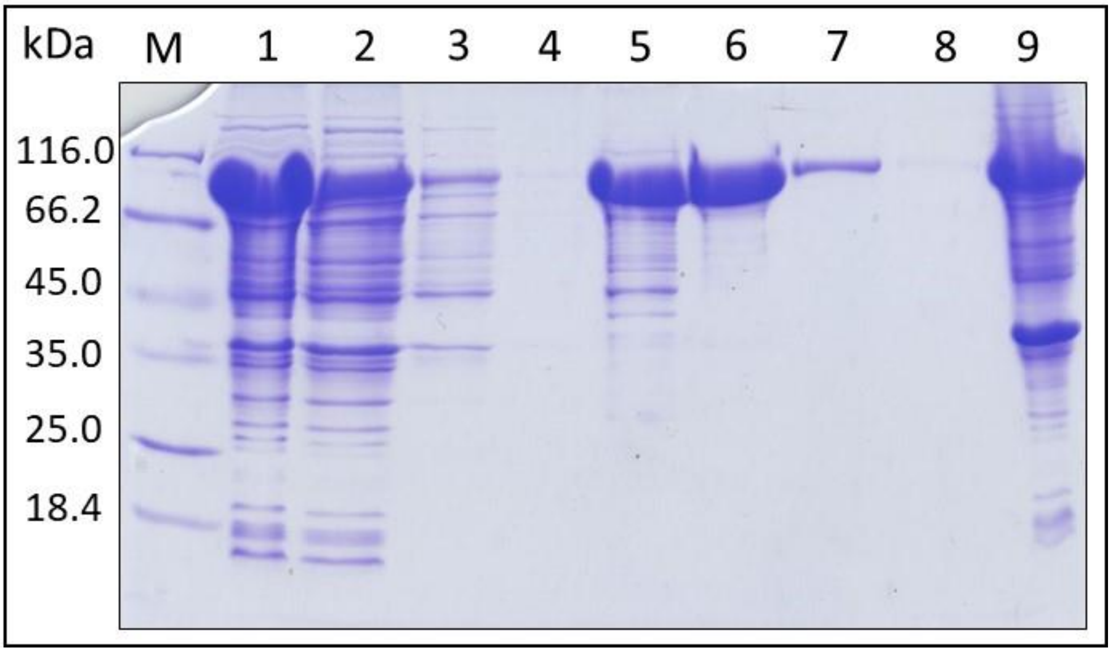

Figure S1: Purification of recombinant AcnB

The gene *acnB* from *Synechocystis* 6803 was cloned as His-tagged version in the expression plasmid pET28a. Recombinant AcnB protein (93.6 kDa) was purified from *E. coli* strain BL21 (DE3) by affinity purification on Ni-NTA columns using the fused N-terminal His-tag. Proteins form different fractions of the purification procedure were separated by SDS-PAGE and stained with Coomassie-brilliant blue. (1- total cell extract, 2 - flow through, 3 - Wash 1, 4 - Wash 2, 5- Wash 3 containing 80 mM imidazole, 6 - Elution 1, 7 – Elution 2, 8 - Elution 3, 9 – Pellet fraction with insoluble proteins; M – Protein size marker, Thermo Fisher Scientific).

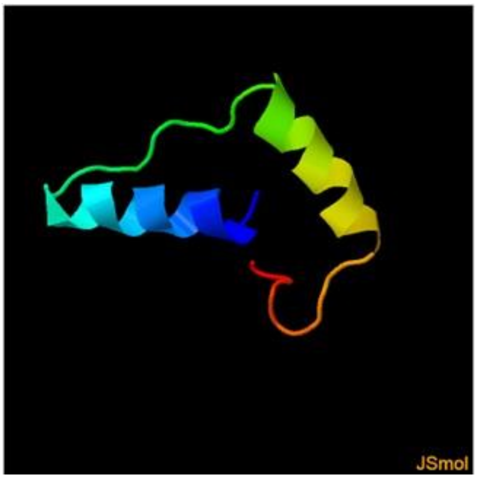

Figure S2: Structure prediction of AcnSP.

The amino acid sequence of AcnSP was sent to the Phyre2 web portal for protein modeling, prediction and analysis (Kelley et al. 2015). The sequence is colored by rainbow from the N-terminal to the C-terminal end. The modeled structure is similar to the N-terminal domain of Aconitase B (d1|5ja1) with 99.6% confidence.

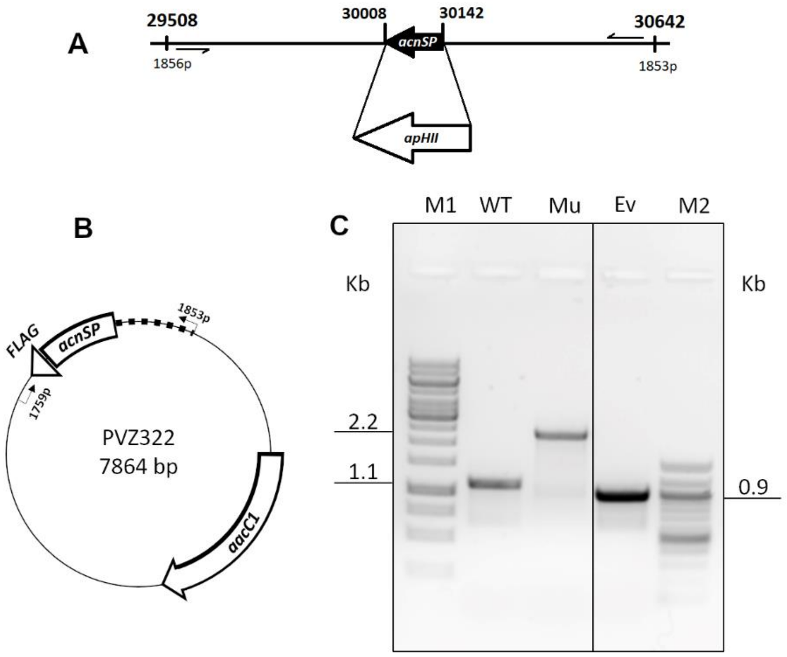

Figure S3: Genotyping of *Synechocystis* 6803 mutant strains

A. Scheme of mutated region of *acnSP* on pSYSA with the location of binding sites for primers 1853p/1856p 5' and 3' of the coding region. These primers are expected to produce fragments of 1113 bp for the WT or 2231 bp for the Δ*acnSP* mutant (KO). **B.** Scheme of plasmid pVZ322 harbouring the FLAG-tagged version of *acnSP*. Binding of primers 1853p and 1759p are expected to produce a fragment of 959 bp. **C.** Genotyping via PCR (lanes: M1 - 1 kb DNA marker, M2 - 100 bp DNA-marker, WT – DNA from wild type, KO – DNA from mutant Δ*acnSP*, Ev – DNA from expression vector pVZ322_acnSP.

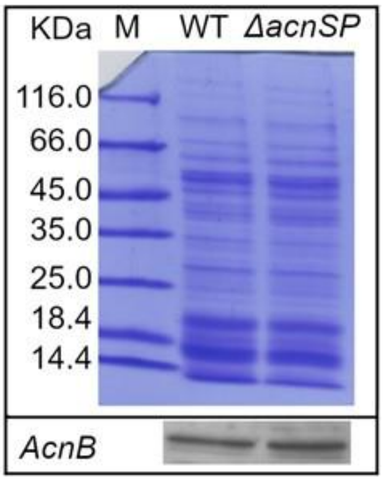

Figure S4: Aconitase abundance in cells growing at 100 µmol photons m^{-2} s^{-1}.

The upper panel shows the Coomassie-stained gel in which 10 µg of total protein extracted from cells of the mutant ∆*acnSP* or the WT were separated by SDS-PAGE. The similar colour intensities indicate equal protein loading. The lower panel shows the detection of aconitase bands in the same protein extracts with anti-AcnB serum. Specific signals between 100 and 80 kDa of similar intensity were obtained.

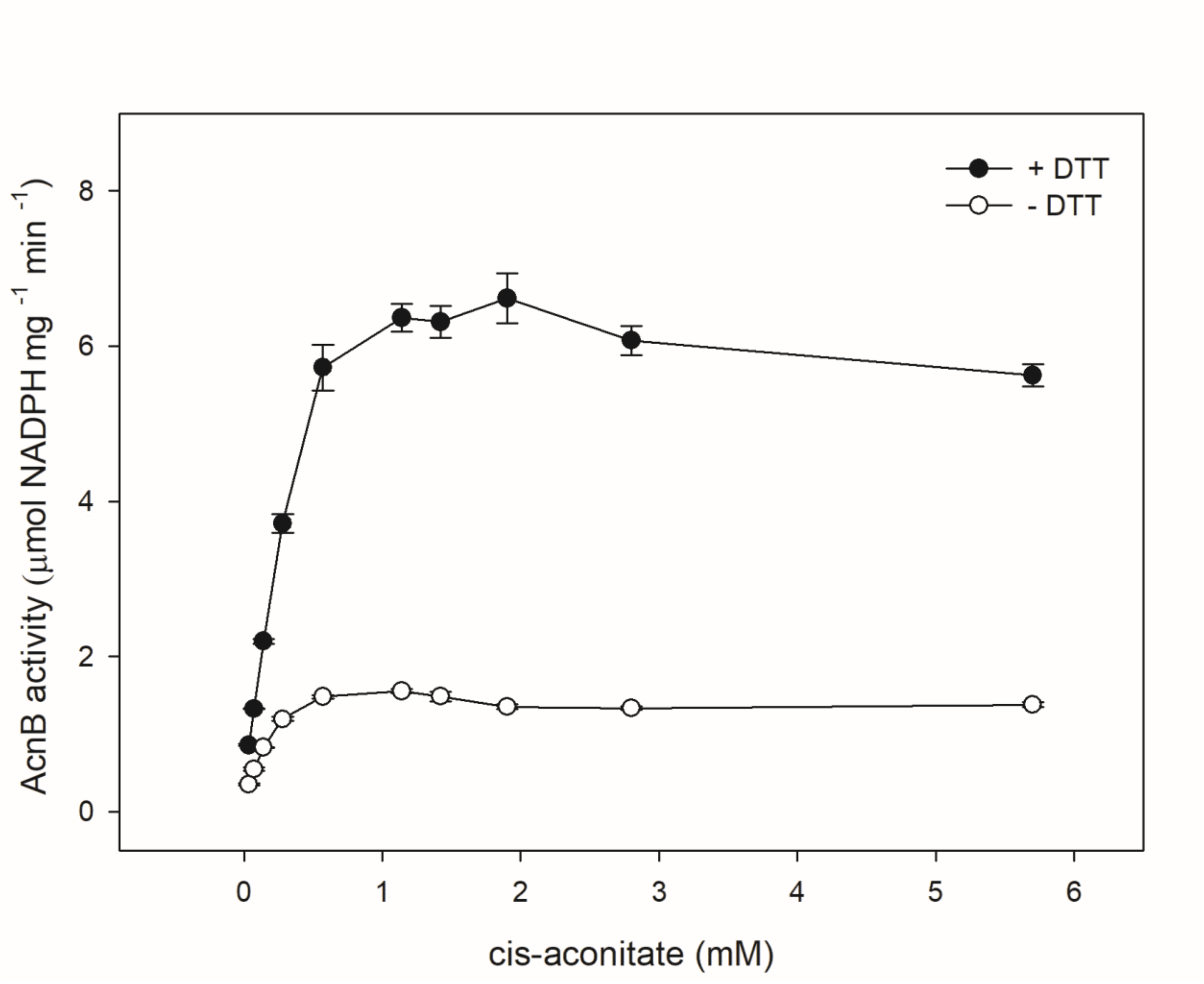

Figure S5: Biochemical characterization of aconitase activity.

Aconitase activity shows Michaelis-Menten kinetics. The aconitase activity was measured with different amounts of substrate (0.02 to 5.7 mM) in the presence or absence of DTT (10 mM final concentration).

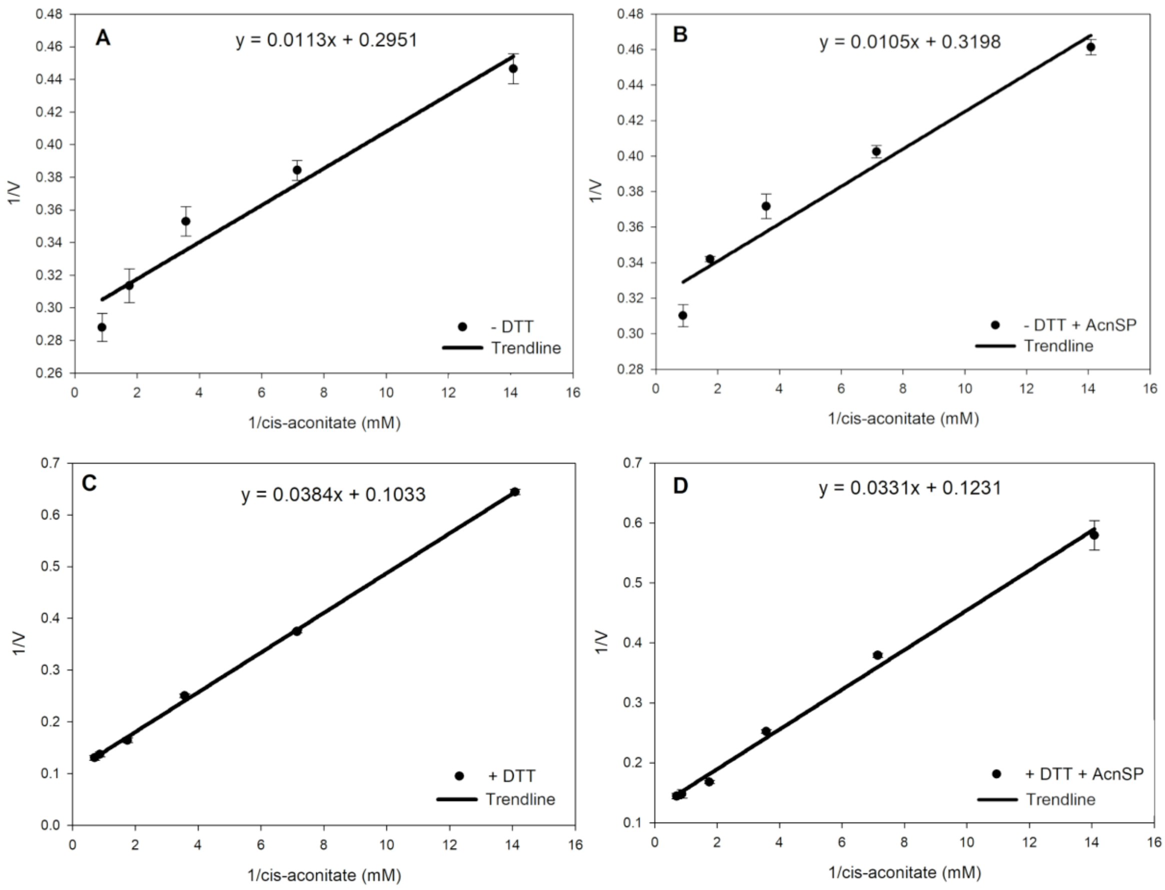

Figure S6: Impact of AcnSP on aconitase activity.

Lineweaver-Burk plots were calculated from aconitase measurements in the presence or absence of AcnSP. The plots were used to estimate kinetic parameters of aconitase. The aconitase activity was measured with different amounts of substrate (0.02 to 2 mM) in the presence (+DTT) or absence (-DTT) of DTT (10 mM final concentration). Approximately 2 nmol aconitase was used per enzyme assay, which were also supplemented with the same amount of the synthetic AcnSP peptide (+AcnSP). Equations and graphs were obtained with the SigmaPlot Software.

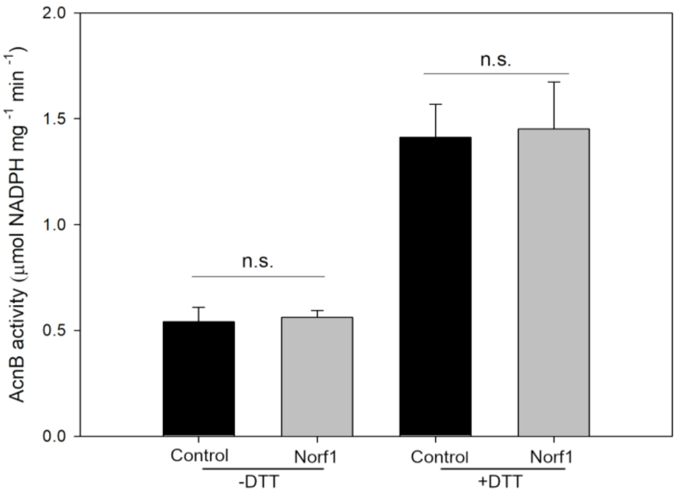

Figure S7: Impact of another small protein Norf1 on aconitase activity.

The aconitase activity was measured at 30 ºC under non-saturating substrate conditions (0.3 mM cis-aconitate, near the estimated K_m value of AcnSP) in the presence or absence of DTT (10 mM final concentration). Approximately 2 nmol recombinant *Synechocystis* 6803 aconitase was used per enzyme assay (control), which were also supplemented with the same amount of the synthetic Norf1 peptide (Norf1).

References for supplementary material

Kelly LA, Mezulis S, Yates CM, Wass MN, Sternberg MJE. 2015. The Phyre2 web portal for protein modeling, prediction and analysis. Nature Protocols **10,** 845-858.
